# Evaluation of the Safety and Efficacy of a Multienzyme Complex in Patients with Functional Dyspepsia: A Randomized, Double-Blind, Placebo-Controlled Study

**DOI:** 10.1089/jmf.2017.4172

**Published:** 2018-11-15

**Authors:** Muhammed Majeed, Shaheen Majeed, Kalyanam Nagabhushanam, Sivakumar Arumugam, Anurag Pande, Mahesh Paschapur, Furqan Ali

**Affiliations:** ^1^ Sami Labs Limited, Bangalore, Karnataka, India.; ^2^ Sabinsa Corporation, East Windsor, New Jersey, USA.; ^3^ Sabinsa Corporation, Payson, Utah, USA.; ^4^ ClinWorld Private Limited, Bangalore, Karnataka, India.

**Keywords:** *digestive enzymes*, *dyspeptic symptoms*, *functional dyspepsia*, *multienzyme complex*, *nonulcer dyspepsia*, *safety and efficacy*

## Abstract

Functional dyspepsia (FD) is a highly prevalent disorder having nonspecific symptoms and varied pathophysiology. Its treatment remains a challenge as therapeutic options are limited, unsatisfactory, and elusive. Thus, safety and efficacy of DigeZyme^*®*^, a proprietary multienzyme complex (MEC), was evaluated as a dietary supplement in FD patients. In this randomized, double-blind, placebo-controlled, parallel-group study, 40 patients were randomly assigned (1:1 ratio) to receive either MEC (50 mg, TID; *n* = 20) or placebo (*n* = 20) for 60 days. Reports of adverse or serious adverse events (AEs), abnormal results of vital signs, abnormal findings during physical examination, and abnormal laboratory investigations were monitored closely. Efficacy measures were change in Short-Form Leeds Dyspepsia Questionnaire (SF-LDQ), Nepean Dyspepsia Index-Short Form (NDI-SF), Visual Analog Scale (VAS), Clinical Global Impression Severity Rating Scale (CGI-S), and Glasgow Dyspepsia Severity Score (GDSS) at baseline and follow-up visits on day 15, 30, and 60. Supplementation with MEC was associated with statistically significant differences (*P* value ranging from .0401 to .0033) in all efficacy parameters compared with placebo. The between-group comparison also revealed that MEC supplement had a significantly greater effect (*P* < .001) versus placebo. No investigation product-related AEs were reported. There were no clinically significant abnormalities in physical findings and no statistically significant changes in biochemical and hematological parameters, vital signs, body weight, and body mass index observed between the two groups at baseline and follow-up visits. MEC supplementation represents an effective and safe alternative to manage dyspepsia symptoms in FD patients.

## Introduction

Dyspepsia, by definition, is not a single symptom but a constellation of symptoms, such as bloating, early satiety, postprandial fullness, nausea, anorexia and heartburn, regurgitation, and burping.^1^ Several reports suggest that majority of dyspepsia cases are diagnosed with minor abnormalities of uncertain significance or an entirely normal endoscopy.^2^ Hence, in the absence of a clinically identifiable structural lesion, it is termed as functional dyspepsia (FD), in part, because disturbed gastrointestinal (GI) function is thought to play an influential role in the progression of symptoms.^3^ FD is also referred to as nonulcerdyspepsia and usually affects young adults, with women being affected more often than men.^4^

Across the globe, FD is a major GI disorder with high prevalence and the most common cause of dyspeptic symptoms, accounting for >70% of dyspepsia cases.^2^ In India, the prevalence rate is believed to be as high as 30%.^5^ As a result, it has remained an expensive option for both primary care and clinical practice—adversely affecting patients' quality of life. Moreover, several definitions of dyspepsia make it difficult to categorize dyspepsia as a pathologically defined entity, owing to the variability of symptoms, which has led to considerable confusion in the literature. However, the Rome criterion is widely accepted for the diagnosis of dyspepsia.^6^

Although accumulating data suggest that infections and possibly food may play an important role in a subset of individuals, at present, the pathophysiology of FD is only partially elucidated. However, there is a growing body of evidence suggesting that FD is, in fact, a very heterogeneous disorder and different mechanisms might be contributing to its onset.^7^ Hence, in clinical practice, FD remains poorly understood with limited options of successful and satisfactory treatment because of the limited availability of pharmacological agents that have demonstrated better efficacy than placebo in randomized controlled trials as well as in the market.^8,9^

According to some physiological studies and case reports, patient education about the possible pathophysiological causes and risk factors associated with FD is the first step involved in the disease management. In addition, lifestyle and dietary recommendations, including avoidance of nonsteroidal anti-inflammatory drugs (NSAIDs), high consumption of coffee, high-fat foods, alcohol, and smoking are also equally important.^9^ Therapeutic intervention includes the use of antacids, prokinetics, H_2_-receptor antagonists, proton-pump inhibitors, *Helicobacter pylori* eradication, herbal preparations, and antidepressants, which are recommended as treatment choice largely by consensus.^9,10^ However, benefits of some treatment approaches have been either disappointing or not satisfactory as results of controlled trials suggest only marginal benefits relative to placebo,^11–13^ symptomatic relief only in a proportion of patients,^14^ inconsistent response rates,^15^ and modest-to-limited efficacy and safety concerns.^16^

Moreover, FD being a disease with heterogeneous pathophysiology, monotherapy may not be suitable for all patients. This has led to the recommendation of a combination of several drugs in different groups of patients.^17^ Thus, efforts are ongoing to identify and develop newer, suitable, and effective treatment options. Additionally, some clinicians believe that clinical experiences appear to support the use of alternative remedies, which is evident from the outcome of several well-designed clinical trials, testing herbal preparations in patients suffering from FD.^18,19^ Digestive enzymes have also been reported to be used for the management of FD.^10,17^

Digestive enzymes, such as amylase, protease, and lipase are produced and secreted by the GI system that aid in digestion by facilitating the breakdown of larger molecules present in food, such as carbohydrates, proteins, and fats, respectively, followed by absorption of nutrients.^20^ Deficiency in digestive enzymes is also believed to be one of the contributing factors for FD, although the possible role of enzyme deficiency in its etiopathogenesis remains unclear.^17,21^ However, a few studies have suggested that therapy with multienzyme preparations is beneficial for reducing symptoms of flatulence, bloating, belching, fullness, and postprandial distress in patients with FD.^22,23^ In another study, Suarez *et al.* demonstrated that pancreatic supplements reduced high-fat meal-related postprandial symptoms in healthy subjects, indicating that enzyme supplementation might be helpful in attenuating FD and related symptomatic responses.^24^ However, clinical studies demonstrating the safety and therapeutic benefits of digestive enzyme complex supplementation in FD patients are not adequate.

Therefore, we aimed the current study to investigate the safety and efficacy of DigeZyme^*®*^, a proprietary multienzyme complex (MEC), in comparison with placebo as a dietary supplement in the management of FD.

DigeZyme^*®*^ is a combination of five digestive enzymes (*α*-amylase, protease, cellulase, lactase, and lipase) that help break down carbohydrates, complex proteins, cellulosic fibers, lactose, and fats. The product is present in the market as a dietary ingredient under the trade name DigeZyme^*®*^ for more than a decade now and has self-affirmed Generally Recognized As Safe status in the United States. In a recent study, MEC was able to decrease delayed onset muscle soreness-associated pain and tenderness in healthy volunteers.^25^

## Materials and Methods

### Study participants

Patients with a medical history and symptoms of FD were identified at the Sparsh Hospital, Bangalore, India from October 2015 to January 2016, who met all the inclusion criteria were enrolled in the study. Participants were selected among patients with following inclusion criteria: (1) subjects from both genders 18–75 years of age, (2) individuals with FD, who fulfilled diagnostic criteria for FD based on the Rome III Diagnostic Criteria, (3) willingness to provide written informed consent and to follow the required protocol, (4) willingness to complete study questionnaires, (5) agree not to use any medication (prescription as well as over-the-counter), including vitamins and minerals during the study period, and (6) not having taken antibiotics or other drugs whose primary site of action is in the GI tract for a period up to 1 month before the beginning of the study.

Exclusion criteria were: (1) pregnancy and breastfeeding, (2) any clinically significant medical history or condition that could jeopardize subject's safety and impact validity of the study results or interfere with the completion of study according to the protocol, (3) organic GI or systemic diseases, diabetes mellitus, and cardiovascular problems, (4) participation in a concurrent trial, (5) use of drugs that interfere with GI motility, (6) history of hypersensitivity reactions, (7) alcoholism or drug abuse in the past 1 year, smoking, or consumption of tobacco products, (8) previous abdominal surgery (except appendectomy), and (9) patients on any therapy, such as homeopathy, ayurvedic *etc*.

The study protocol was reviewed and approved by the Ethics Committee of Sparsh Hospital. The study was conducted according to the guidelines laid down in the Declaration of Helsinki (Edinburgh, 2000) and the ICH-harmonized tripartite guidelines regarding good clinical practice, and all participants provided a written informed consent. The trial has been registered in the Clinical Trial Registry India (http://ctri.nic.in/Clinicaltrials/pmaindet2.php?trialid=12637&EncHid=&userName=functional%20dyspepsia%20enzyme%20complex).

### Investigation product

The MEC used in the present study was supplied by Sabinsa Corporation, NJ, USA. Participants were administered hard gelatin capsules, each containing 50 mg of MEC. Selection of dose for this study was based on a review of safety data of individual enzymes and the blend. Placebo capsules were matched with respect to size and shape and contained the equivalent weight of maltodextrin.

### Study design

This study was designed as a randomized, double-blind, placebo-controlled, parallel-group trial. Initially, patients underwent screening procedures comprising of assessment of demographic data, medical history, and medication history; patients were subjected to physical examination, vital signs, and blood sample for laboratory analysis; and women of child-bearing age were required to have a negative urine pregnancy test during their participation in the study. To identify cases of *H*. *pylori*, stool samples were tested for *H*. *pylori* infections, however, patients with the diagnosis of *H. pylori* infection were not excluded from the study. Endoscopy was performed to exclude patients with gastroesophageal reflux disease (GERD), irritable bowel syndrome (IBS), and other chronic GI disorders. Concurrent medications, if any, were recorded.

After acceptance for inclusion in the trial, enrolled subjects visited the clinic on day 0 (baseline/visit 1), which was between 5 to 7 days from the day of screening, wherein they were instructed on their daily dose of study supplement. The subjects were blinded and received dosing as per randomization code provided at the site by an authorized person independent of the study center. They were randomly allocated in a 1:1 ratio and were instructed to take three capsules (50 mg each) of either MEC or placebo daily as a dietary supplement for a period of 60 days and were allowed to consume their regular diet. Subsequent visits were on day 15 (visit 2), day 30 (visit 3), and day 60 (visit 4/final). A follow-up visit was arranged (15 days from the final visit) to inquire patients on the incidence of adverse events (AEs) if any, since his/her last visit, and overall general wellbeing.

### Assessment of safety and efficacy outcomes

#### Safety outcomes

Safety was assessed by the analysis of any reports of adverse or serious AEs, abnormal results of vital signs, abnormal findings during physical examination, and abnormal results from laboratory investigations that were done at the screening/baseline and were repeated during each visit until the study concluded. All adverse experiences were rated by the study investigator for intensity and relationship to the study product if any.

#### Efficacy outcomes

The efficacy was assessed using different questionnaires at every visit starting from baseline until the final visit, which included: Short-Form Leeds Dyspepsia Questionnaire (SF-LDQ) (for the assessment of individual symptoms based on frequency and severity),^26,27^ Nepean Dyspepsia Index–Short Form (NDI-SF) (for the assessment of quality of life),^28^ Visual Analog Scale (VAS) (for the assessment of pain),^29^ Clinical Global Impression Severity Rating Scale (CGI-S) (for the assessment of severity of illness),^30^ and Glasgow Dyspepsia Severity Score (GDSS) (to measure the global and personal impact of dyspeptic symptoms).^31^ Except for CGI-S, all others were administered as self-completion questionnaire by the patients, whereas CGI-S was administered by the physician measuring the frequency and severity of dyspepsia symptoms.

The efficacy outcome measures included the change in the efficacy assessment (*i.e*., the difference between scores of SF-LDQ, NDI-SF, VAS, CGI-S, and GDSS from baseline to the final visit).

### Statistical analysis

SAS version 9.2 was used for data analysis. Paired “*t*” test, analysis of covariance (ANCOVA), and Wilcoxon signed rank sum test were used for appropriate data set variables to reach the best possible statistical conclusion between the MEC- and placebo-receiving groups. The baseline descriptors were summarized as mean and standard deviations for continuous variables and as frequencies and percentages for categorical variables. Last Observation Carry Forward (LOCF) method was followed for efficacy evaluations of subjects whose data were not available in the final visit.

## Results

### Safety

**FIG. 1.**
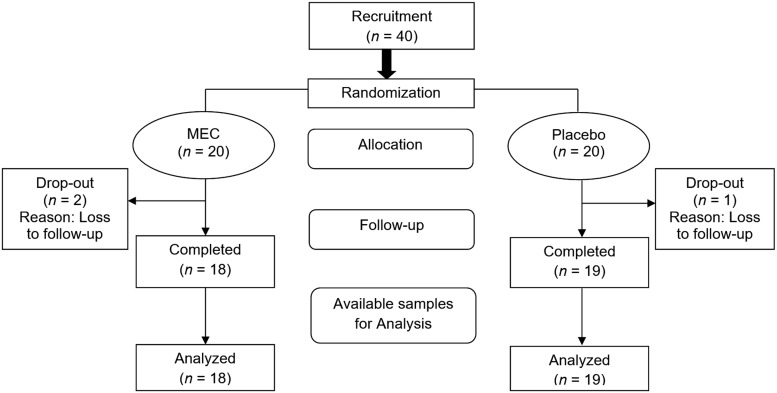
Flowchart of the study procedures.

Forty subjects (8 male and 32 female) fulfilled the inclusion criteria and were entered into the trial. Patients were assigned to receive either MEC (*n* = 20) or placebo (*n* = 20). Two subjects dropped out from the MEC group (citing personal reasons, the patients did not come for follow-up visits), whereas one withdrawal was observed in the placebo group (patient was not willing to come for follow-up visits). Overall, 37 subjects (*n* = 18; MEC group and *n* = 19; placebo group) completed the study with good compliance (Fig. 1). No AEs were reported pertaining to the product under investigation. Although there were five AEs, viz. severe stomach pain, excess bloating, pain while passing the stools, abdominal pain, and increase in bloating reported in the placebo group, the investigator classified these AEs as having no possible relationship with the treatment and events resolved without the use of any concomitant medication.

Demographic characteristics of study subjects were recorded on the day of screening (Table 1). Except for FD, none of the subjects was having an abnormal medical history at the time of enrollment. There were no clinically significant abnormalities in physical findings as well as a change in the body weight and body mass index values observed between the two groups from baseline to follow-up visits.

**Table 1.** Demographic Characteristics at Baseline

*Parameter*	*Values (range)*
Age (years)	42 ± 11.12 (19–65)
Height (cm)	157.4 ± 7.97 (140–171)
Weight (kg)	65.4 ± 13.16 (45–94)
BMI	26.5 ± 5.01 (19–38)

	*n (%)*
Gender	
Male	8 (20)
Female	32 (80)
Tobacco history	
Nonuser	40 (100)
Drinking history	
Nondrinker	40 (100)

All values are expressed as mean ± SD.

BMI, body mass index.

To confirm if the cases presented to the hospital are nonulcer dyspepsia and to rule out GERD, IBS, and other chronic GI diseases, endoscopy was performed on two screened patients. To ascertain whether cases were related to infections with *H*. *pylori* or were independent, stool analysis was performed for all subjects during the screening visit. The test results showed that 92.5% (*n* = 37) patients were not infected with *H*. *pylori*.

No statistically significant changes were observed in vital signs, such as blood pressure (systolic and diastolic), pulse rate, respiratory rate, and heart rate between the two groups at baseline and at the end of the study (Table 2), as well as during all follow-up visits (data not shown). Similarly, biochemical parameters were within the normal range with no significant changes for both the groups (Supplementary Table S1; Supplementary Data available online at www.libertpub.com/jmf).

**Table 2.** Effect of Multienzyme Complex Supplementation on Vital Signs Before and After Treatment

*Parameter (units)*	*Visit*	*MEC*	*Placebo*	P
Systolic BP (mmHg)	Baseline	127.0 ± 8.65	122.0 ± 8.34	.70
	Final	125.6 ± 6.16	124.2 ± 8.38	.44
Diastolic BP (mmHg)	Baseline	78.5 ± 8.13	80.5 ± 6.86	.63
	Final	77.2 ± 4.61	78.4 ± 6.88	.33
Heart rate (beats/min)	Baseline	73.3 ± 2.99	74.4 ± 2.64	.95
	Final	73.6 ± 2.01	72.9 ± 2.44	.08
Pulse rate (beats/min)	Baseline	73.3 ± 2.99	74.4 ± 2.64	.95
	Final	73.6 ± 2.01	72.9 ± 2.44	.08
Respiratory rate (breaths/min)	Baseline	21.3 ± 1.68	20.4 ± 1.19	.88
	Final	21.2 ± 1.38	20.8 ± 1.86	.43

All values are expressed as mean ± SD.

BP, blood pressure; MEC, multienzyme complex.

### Efficacy

Treatment compliance was satisfactory with 75% of the population meeting >95% compliance. Adherence to therapy was assessed by pill count and participants' self-report during each follow-up visit.

**FIG. 2.**
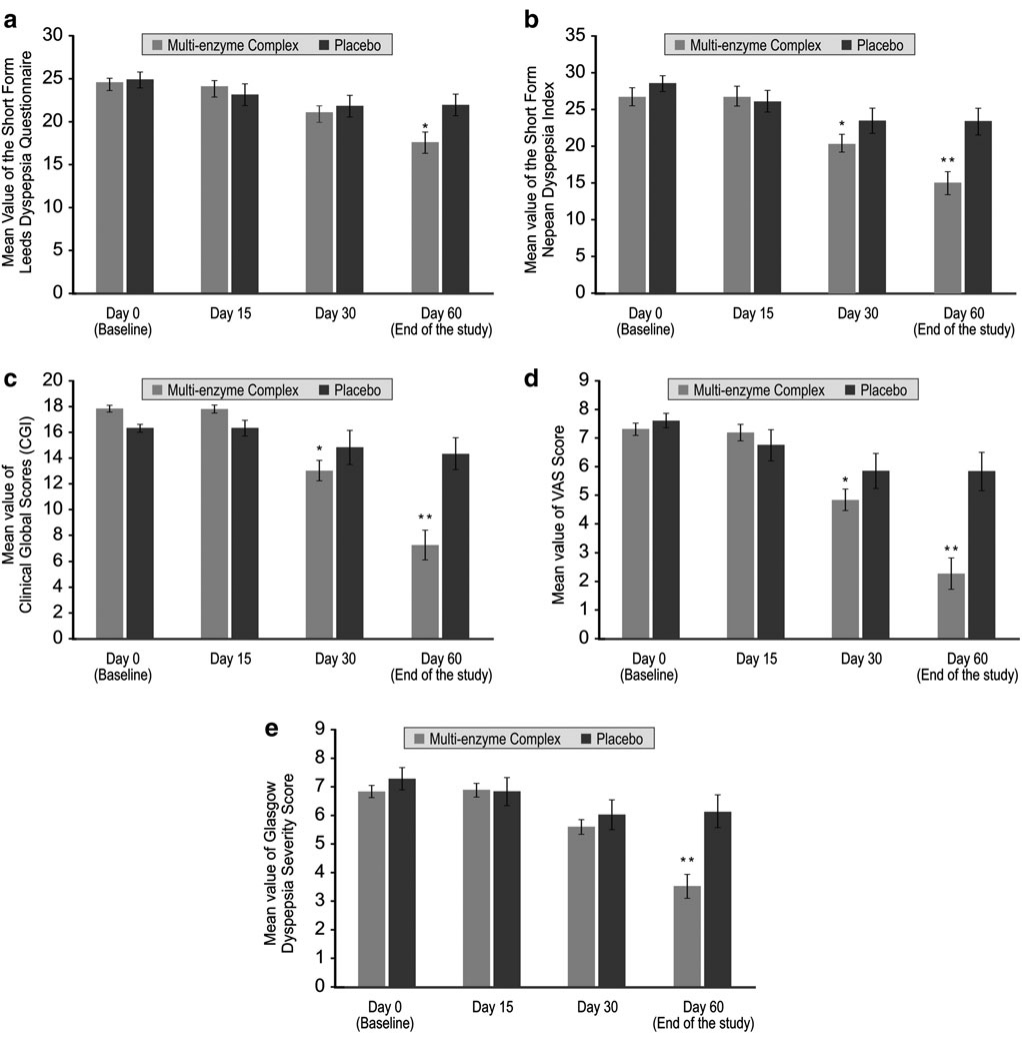
Efficacy measures at baseline, 15, 30, and 60 days (end of the study). All the values are expressed as mean ± SE. **(a)** Short-Form Leeds Dyspepsia Questionnaire, **(b)** Short-Form Nepean Dyspepsia Index, **(c)** CGI Scores, **(d)** VAS score, and **(e)** Glasgow Dyspepsia Severity Score. *P < .01 between the treatment groups and also between baseline and end of the study (day 60). **P < .001. CGI, clinical global impression; VAS, visual analog scale.

In the efficacy assessments, comparative mean values of MEC and placebo groups between baseline and end of the study are presented for SF-LDQ, NDI-SF, CGI-S, VAS, and GDSS (Table 3). Between-group comparisons revealed that MEC supplementation showed a significantly greater effect (*P* < .01) versus placebo when their respective values at the end of the study were analyzed. Additionally, statistical analysis revealed that all efficacy measures were statistically significant at day 30 and maintained the same up to end of the study (day 60) (Fig. 2b–d), except SF-LDQ and GDSS, which also reached a statistically significant level at day 60 (Fig. 2a, e).

**Table 3.** Effect of Multienzyme Complex Supplementation on Evaluated Efficacy Assessments

	*MEC*		*Placebo*		P	
*Parameters*	*Baseline*	*Final visit*	*Baseline*	*Final visit*	*Within group*	*Between groups*
SF-LDQ	24.5 ± 3.15	17.9 ± 4.87	24.9 ± 3.92	21.5 ± 5.95	.0401	<.01
NDI-SF	26.7 ± 5.73	15.7 ± 6.79	28.5 ± 4.73	22.7 ± 8.28	.0115	<.01
CGI-S	17.8 ± 1.12	8.3 ± 5.73	16.3 ± 4.06	13.8 ± 5.91	.0049	<.01
VAS	7.3 ± 1.22	2.6 ± 2.48	7.6 ± 1.10	5.6 ± 3.12	.0033	<.01
GDSS	6.8 ± 1.28	3.8 ± 1.91	7.3 ± 1.74	5.8 ± 2.78	.0159	<.01

All values are expressed as mean ± SD.

CGI-S, Clinical Global Impression Severity Rating Scale; GDSS, Glasgow Dyspepsia Severity Score; NDI-SF, Nepean Dyspepsia Index–Short Form; SF-LDQ, Short-Form Leeds Dyspepsia Questionnaire; VAS, Visual Analog Scale.

To check the effect of the baseline values as potential cofounders on the observed associations, ANCOVA was performed. According to the results, MEC group showed a statistically significant difference (*P* value ranging from .0401 to .0033) in all efficacy parameters compared with the placebo group. Symptom severity scores on the SF-LDQ, a reliable, valid, and responsive outcome measure for quantifying the frequency and severity of dyspepsia symptoms, improved significantly (*P* = .0401) at the end of the study in MEC group.

Data from SF-LDQ indicated that MEC group had a significant reduction in the symptoms of epigastric pain, postprandial distention, indigestion, heartburn, and nausea, whereas the placebo group showed no such improvement. Similarly, at the end of the study (*i.e*., on day 60), NDI-SF scores were better among subjects who were supplemented with MEC than among those who received placebo. The NDI-SF score improved by a mean of 15.7 ± 6.79 (vs. 26.7 ± 5.73 at baseline) with MEC (*P* = .0115), whereas in the placebo group it was 22.7 ± 8.28 (vs. 28.5 ± 4.73 at baseline). Results of the NDI-SF questionnaire, a valid, disease-specific index used to measure symptoms and health-related quality of life in FD patients, thus suggest that supplementation with MEC significantly improved the quality of life of subjects with FD at the end of the study.

Assessment of severity of illness using CGI-S also showed that the active group markedly improved from baseline to final visit (*P* = .0049), whereas no such improvement was observed in the placebo group. Assessment of VAS suggested that at the end of the study MEC supplementation showed a significant improvement (2.6 ± 2.48) with respect to baseline values (7.3 ± 1.22) of all the evaluated GI symptoms (*P* = .0033), whereas the placebo group showed no significant changes (5.6 ± 3.12 vs. 7.6 ± 1.10 at baseline). Based on the VAS analysis it can be said that MEC was efficacious in changing or decreasing GI symptoms such as postprandial fullness, early satiety, bloating, epigastric discomfort, epigastric pain, postprandial nausea, belching after meals, and vomiting in the active group on day 60. Results from GDSS questionnaire, a tool for the global measurement of dyspepsia, also implied that at the end of the study, patients receiving MEC had less severity of dyspepsia and better response to the supplementation than at baseline as well as the placebo group.

Overall, our results are in agreement with findings of previous studies,^17,22,23,32^ wherein significant efficacy was demonstrated by multienzyme preparations in alleviating frequency and severity of dyspepsia symptoms and patients had shown better tolerability.

## Discussion

Results of the present trial supported the effectiveness of MEC as a dietary supplement in relieving the symptoms associated with FD, as evidenced by marked improvement in all assessed efficacy measures. MEC was safe and well tolerated during the trial with no reported AEs pertaining to the investigation product.

FD is a clinical problem of considerable magnitude because of its high prevalence rate, and the chronic and recurrent nature of symptoms, whose management is a challenge for gastroenterologists. In addition to that, the therapeutic options are limited and subprime; pharmacological therapies have often failed,^16,33–37^ or shown cardiac toxicity potential,^16,38–41^ and were not recommended for various reasons.^7,42,43^

Although recent advances have improved our understanding of the pathophysiology of FD, the advances have yet to result in new safe and highly effective treatment options.^44^

Since varied changes in GI function are associated with meal digestion, followed by potential pathophysiological mechanisms, many FD patients have reported exacerbation of symptoms following food ingestion, particularly high-fat-containing meals.^45^ In a double-blind, crossover study, a combination of digestive enzymes, such as lipase, protease, and amylase reduced the postprandial symptoms, such as bloating, gas, and fullness after ingestion of a high-calorie, high-fat meal in healthy volunteers.^24^ Furthermore, epidemiological studies in the United States and Europe have also shown that FD-related symptoms are meal associated in 50–80% of the population.^46^ Since a transient deficiency in digestive enzymes has been known to have a causal relationship with FD, oral digestive enzymes are often prescribed to patients complaining of various dyspeptic symptoms.^17^ The rationale for prescribing digestive enzyme supplements could possibly be related to the fact that various digestive enzymes play a crucial role in breaking down several complex carbohydrates, fats, and proteins into smaller units, which are then assimilated. Hence, it can be postulated that supplementing with digestive enzymes in FD patients having dyspeptic symptoms may aid in the digestive process and in turn, alleviate symptoms associated with undigested and poorly absorbed nutrients.

Oral supplementation of digestive enzymes has stability issues. However, certain plant- and microbe-derived enzymes are known to be stable under a broad pH and temperature range and hence they are capable of acting throughout the human GI tract without being affected by the gastric secretions.^47^

Supplemental enzymes, mainly of plant and fungal origin, interact with undigested foods in the upper region of the stomach (pH 4–6.5) for ∼1 h before coming in contact with gastric secretions (hydrochloric acid and pepsin) at the lower portion of the stomach, where the actual digestive process takes place. This is termed as “predigestion.” Hence, enzyme supplements may withstand denaturation and hydrolysis by the gastric secretions through the “predigestive” process.^48^

Moreover, few enzymes would survive in the pure gastric environment (pH 1.5–4) as the presence of food in the lower part of the stomach buffers the gastric pH considerably (ranging from 2.5 to 5, based on the type of food consumed). It has been hypothesized that although enzymes might become “temporarily” inactive or denatured because of low pH-induced unfolding in the gastric environment, they may recuperate their enzymatic activity once they reach the intestine, where pH would be ranging from 7 to 8.5. In fact, many enzymes are believed to function optimally under highly acidic conditions in the stomach.^47,48^

Additionally, numerous studies have demonstrated gastric survivability of multienzyme formulations containing lipase, protease, amylase, lactase, and cellulose from bacterial and/or fungal origin.^48–52^

Enzymes present in the MEC used in the current study are from bacterial (protease is from *Bacillus subtilis*) and fungal origin (*α*-amylase and lactase from *Aspergillus oryzae*; lipase from *Rhizopus oryzae*, and cellulase from *Trichoderma longibrachiatum*), and are produced by the fermentation process. Based on the available literature cited above, we strongly believe that the MEC would sustain harsh gastric environment upon oral administration.

In recent years, several well-designed clinical studies have shown that pancreatic or digestive enzyme supplements could be promising alternative approaches in managing FD syndrome.^10,22,53^ Postmarketing surveillance studies of the multienzyme formulation to evaluate the efficacy and tolerability revealed that treatment was able to decrease frequency and severity of various dyspeptic symptoms in FD patients.^23^ In another multicenter, randomized, placebo-controlled, crossover study, treatment with an enzyme preparation containing *Aspergillus oryzae* extract (cellulase, protease, and amylase) and pancreatin (lipase, proteinase, and amylase) in patients diagnosed with chronic digestive disorders, including FD, showed a significant reduction in the severity index of dyspeptic symptoms compared with placebo treatment.^32^ In clinical practice, apart from treating pancreatogenic steatorrhea or use in chronic pancreatitis-associated pain management, exogenous pancreatic enzymes have also been used in FD patients.^54^

Prolonged postprandial symptoms of fullness and abdominal discomfort are common in FD patients after meals. Previous studies have demonstrated that meals with high-fat content delayed gastric emptying, causing bloating, and prolonged the sensation of stomach fullness in healthy volunteers, which are typical postprandial symptoms experienced after normal meals by FD patients. These symptoms were significantly reduced upon enzyme supplementation.^24,55^ Although digestive enzyme deficiency has been linked to causing dyspepsia, the exact mechanism involved is not clearly known because of the frequency and multiplicity of the etiopathogenesis of enzyme deficiency. However, based on the reported observations^17,55,56^ and efficacy outcomes of the current study, we hypothesize that digestive enzyme supplementation enhances the normal actions of digestive enzymes during the gastric phase of food digestion, which in turn results in decreased varying dyspepsia symptoms in FD patients.

Normal gastric neuromuscular activity is essential for mixing and emptying stomach contents. Pilichiewicz *et al.* demonstrated a significant correlation between the FD symptoms and plasma cholecystokinin, suggesting the possible involvement of gut hormones.^45,57^ Hence, we speculate that MEC may be helpful in alleviating at least some of the FD symptoms (*e.g*., delayed gastric emptying) by enhancing the action of digestive enzymes, especially in conditions such as postprandial distress syndrome. However, further studies are required in this regard to elucidate the definitive physiological mechanism involved.

Ingestion of fatty meals result in a decrease in the normal gastric myoelectrical activity and enhanced tachygastria in healthy individuals.^55^ Interestingly, similar observations were made by Pfaffenbach *et al.* previously in patients diagnosed with FD, wherein patients demonstrated a significant increase in tachygastria preprandially compared with the control group. Furthermore, FD patients with delayed gastric emptying showed significantly more pre- and postprandial tachygastria when compared with patients having normal gastric emptying.^58^ Hence, based on earlier study findings^55,56^ and current study observations, it would be possible that enzyme supplementation may provide relief to FD patients by controlling some of the common dyspeptic symptoms. However, further studies are warranted in this regard to delineate the underlying mechanism. Additionally, the safety data of the study concluded that MEC supplementation produced no significant changes in the biochemical and hematological parameters and vital signs from the screening/baseline to the end of the study.

Although a number of studies have demonstrated the beneficial role of digestive enzyme preparations in alleviating dyspepsia-related symptoms, we believe that the current study findings are novel in several aspects. MEC used in the present study is a unique combination of five enzymes (lipase, protease, *α*-amylase, lactase, and cellulose) of fungal and bacterial origin, whereas most of the reported studies have used either different enzyme combinations of both animal and microbial origin,^17,23,32^ in combination with drugs or micronutrients,^17,22,59^ or standalone enzyme preparations.^56^ Furthermore, to the best of our knowledge, this study is the first to report on the safety and efficacy of multienzyme preparation, at least having the abovementioned five enzymes of microbial origin, in the management of FD symptoms in the Indian population. Moreover, different self-completion questionnaires for patients (SF-LDQ, NDI-SF, VAS, and GDSS) were also used to determine the effectiveness of MEC, which distinguish the current study from the previous trials, wherein the efficacy was mostly assessed only by physicians. Thus, the current findings will be helpful in further advancing research on the multienzyme preparations in managing the clinical symptoms of patients with FD.

In conclusion, the present study provided clinical evidence supporting the safety and efficacy of MEC as a dietary supplement in the management of dyspeptic symptoms in patients with FD. These findings support the use of digestive enzyme supplements, maybe as an adjuvant therapy.^53^ However, further prospective, larger-scale trials with extended follow-up durations are warranted to establish underlying mechanism as well as a detailed assessment of therapeutic effects of digestive enzyme supplementation in managing dyspeptic symptoms in patients with FD.

## Ethics Approval and Consent to Participate

- The study protocol and related documents were reviewed by the Institutional Ethics Committee, Sparsh Hospital for Advanced Surgeries, Bangalore, which gave a favorable written opinion for the conduct of this study
- The trial has been registered in the Clinical Trial Registry India (http://ctri.nic.in/Clinicaltrials/pmaindet2.php?trialid=12637&EncHid=&userName=functional%20dyspepsia%20enzyme%20complex)

## Supplementary Material

Supplemental data
