# Supplementary material for: Evaluation of the Safety and Efficacy of a Multienzyme Complex in Patients with Functional Dyspepsia: A Randomized, Double-Blind, Placebo-Controlled Study
Source: J Med Food. 2018 Nov 15;21(11):1120–8. doi: 10.1089/jmf.2017.4172 (PMC6249666; doi:10.1089/jmf.2017.4172)
Supplement: Supplemental data [file Suppl_Table1.pdf]

Supplementary Data

Supplementary Table S1. Effect of Multienzyme Complex Supplementation on Biochemical and Hematological Parameters Before and After Treatment

Parameter (units)	*Visit*	*MEC*	*Placebo*	*Normal range*
Albumin (g/dL)	Screening	4.4 ± 0.46	4.3 ± 0.56	3.5–5.2
	Final	4.4 ± 0.23	4.4 ± 0.25	
Alkaline phosphatase (U/L)	Screening	23.5 ± 7.56	27.5 ± 9.92	20–140
	Final	21.0 ± 9.42	19.0 ± 7.08	
Aspartate aminotransferase (U/L)	Screening	21.3 ± 4.54	21.3 ± 5.22	0–40
	Final	20.7 ± 7.02	19.5 ± 4.77	
Blood urea nitrogen (mg/dL)	Screening	9.9 ± 3.84	9.7 ± 3.69	5–24
	Final	10.4 ± 2.44	10.8 ± 2.68	
Creatinine (mg/dL)	Screening	0.7 ± 0.13	0.7 ± 0.10	0.5–1.2
	Final	0.7 ± 0.11	0.7 ± 0.10	
Fasting blood sugar (mg/dL)	Screening	94.6 ± 14.97	88.4 ± 16.01	70–110
	Final	111.7 ± 69.84	101.0 ± 38.40	
LDL cholesterol (mg/dL)	Screening	122.5 ± 30.09	115.6 ± 36.85	100–130
	Final	112.9 ± 20.20	110.5 ± 27.82	
Potassium (mEq/L)	Screening	4.4 ± 0.48	4.4 ± 0.36	3.5–5.2
	Final	4.5 ± 0.52	4.3 ± 0.39	
Sodium (mEq/L)	Screening	142.3 ± 2.06	142.2 ± 2.27	136–145
	Final	140.1 ± 2.20	140.7 ± 1.87	
Total bilirubin (mg/dL)	Screening	0.5 ± 0.22	0.5 ± 0.43	0.1–1.2
	Final	0.4 ± 0.16	0.4 ± 0.28	
Total protein (g/dL)	Screening	7.2 ± 0.70	7.4 ± 0.62	6–8
	Final	7.5 ± 0.76	7.3 ± 0.60	
Erythrocyte count (RBC) ($*10^6$ cells/mm^3)	Screening	5.8 ± 2.85	5.9 ± 3.99	4–6.5
	Final	4.6 ± 0.41	4.6 ± 0.59	
Hematocrit (%)	Screening	38.6 ± 3.81	39.8 ± 3.93	40–50
	Final	37.7 ± 4.31	37.6 ± 5.52	
Hemoglobin (g/dL)	Screening	13.2 ± 1.49	13.5 ± 1.40	11–16
	Final	12.7 ± 1.80	12.6 ± 2.08	
Leukocyte count (WBC) (cells/mm^3)	Screening	9550.0 ± 2270.52	9795.0 ± 1863.35	4000–11000
	Final	10661.1 ± 2553.46	9110.5 ± 2012.71	
Platelet count ($*10^5$/mm^3)	Screening	2.9 ± 0.57	2.9 ± 0.53	1.5–4.5
	Final	3.4 ± 1.00	3.0 ± 0.80	

All values are expressed as mean ± SD.

LDL, low-density lipoprotein; MEC, multienzyme complex; RBC, red blood cells; WBC, white blood cells.
